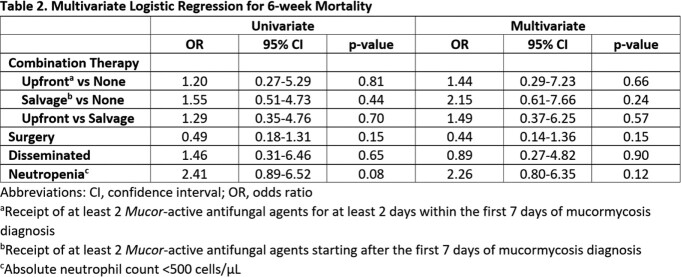# 824. Combination Antifungal Therapy for Invasive Mucormycosis in Immunocompromised Hosts: A Single-Center Experience

**DOI:** 10.1093/ofid/ofad500.869

**Published:** 2023-11-27

**Authors:** Brian Lu, David R Ha, Sa Shen, Jessica Ferguson, Ailin Kim, Sarah Kim, Emily Mui, Stan Deresinski, Marisa Holubar, William Alegria

**Affiliations:** Stanford Health Care - Tri-Valley, Stanford, California; Stanford Health Care, Stanford, California; Quantitative Sciences Unit, Stanford, California; Stanford University, Palo alto, California; Stanford Health Care, Stanford, California; Stanford Health Care, Stanford, California; Stanford Health Care, Stanford, California; Stanford Health Care, Stanford University School of Medicine, Stanford, California; Stanford University School of Medicine, Stanford, CA; Stanford Health Care, Stanford University School of Medicine, Stanford, California

## Abstract

**Background:**

Invasive mucormycosis continues to be associated with high rates of mortality despite advances in management strategies. Recent studies have suggested a benefit to early combination therapy, but overall, the data remain limited regarding its impact on outcomes.

**Methods:**

We conducted a retrospective study from January 2009 to December 2022. We included patients aged 18 years or older with proven or probable invasive mucormycosis based on the European Organization for Research and Treatment of Cancer and the Mycoses Study Group 2020 criteria and a history of hematologic malignancy, bone marrow transplant, or solid organ transplant. The primary outcome was all-cause mortality up to 6-weeks after diagnosis. Combination therapy was defined as receipt of >2 *Mucor*-active antifungals for >3 days and was further categorized as upfront (within 7 days of diagnosis of mucormycosis) or salvage (starting after 7 days post-diagnosis of mucormycosis).

**Results:**

Eighty-two patients met inclusion criteria and were included for analysis. Most patients had a hematologic malignancy (78%) and the most common infection site was pulmonary (62%). Baseline demographics were similar between groups except for the causative organism of invasive mucormycosis. 36 patients (44%) underwent surgical intervention and 58 patients (71%) received combination antifungal therapy. Microbiologically confirmed cases were more common in patients receiving upfront (78%) and salvage combination therapy (50%) compared to monotherapy (34%, p=0.005). Despite being on prophylaxis targeting mucormycosis, 24 patients (29%) developed invasive mucormycosis. After adjusting for surgery, neutropenia, and disseminated disease in multivariate analysis, there was no significant association with either upfront or salvage combination therapy but numerically higher odds of 6-week mortality when compared to monotherapy (OR 1.44, p=0.66 and OR 2.15, p=0.24, respectively).

**Conclusion:**

There was no difference in 6-week mortality for patients receiving antifungal combination or monotherapy for invasive mucormycosis. We observed a numerically higher odds of 6-week mortality in patients receiving combination therapy, which may be explained by confounding by indication.

**Disclosures:**

**All Authors**: No reported disclosures

**Figure:**
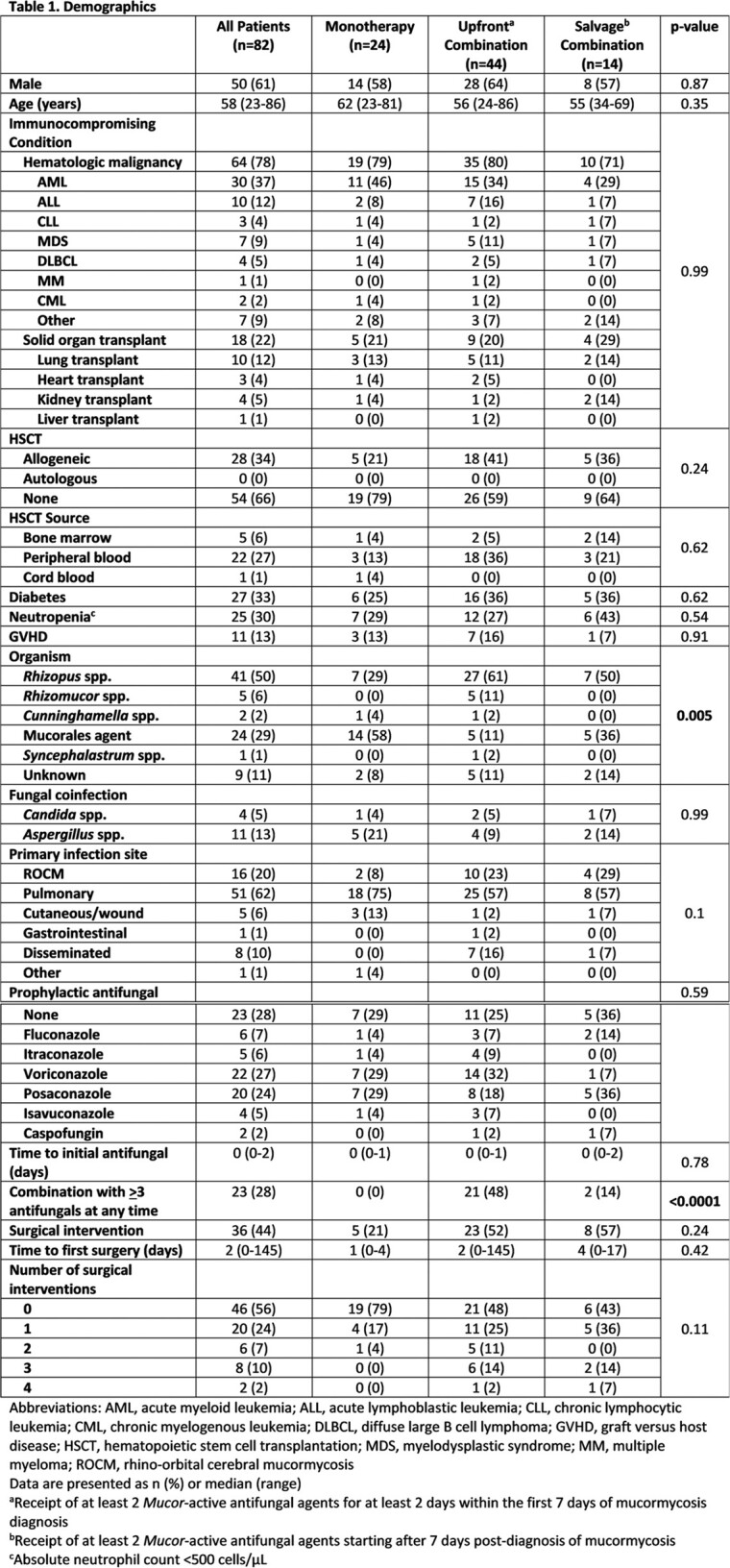

**Figure:**